# Outcomes and experiences of DIALOG+ provided remotely for patients with anxiety disorders—A non-controlled pilot trial

**DOI:** 10.1371/journal.pone.0321744

**Published:** 2025-05-19

**Authors:** Natividad Olivar, Fernando Luis Carbonetti, Luis Ignacio Brusco, Stefan Priebe

**Affiliations:** 1 Center of Behavioral Neurology and Neuropsychiatry, School of Medicine, University of Buenos Aires, Buenos Aires, Argentina; 2 Centre for Psychosocial Medicine, University of Hamburg, Hamburg, Germany; Old Dominion University, UNITED STATES OF AMERICA

## Abstract

**Background:**

Low-cost interventions in routine care are needed to reduce the burden of anxiety disorders. DIALOG+ is an evidence-based intervention specifically designed to make routine patient-clinician meetings in mental health care therapeutically effective. It has been shown to be beneficial in a range of studies, but so far not been tested in patients with a primary diagnosis of anxiety disorders.

**Methods:**

We conducted a non-controlled pilot trial in an out-patient service in Buenos Aires, Argentina. DIALOG+ was applied five times over a six-month period. Because of the pandemic all sessions had to be delivered remotely which had not been originally planned. At baseline and after the intervention, we assessed as outcome criteria subjective quality of life on the Manchester Short Assessment of Quality of Life, general symptoms on the Brief Psychiatric Rating Scale and the objective social situation on the Social Outcomes Index. Patient experiences were explored in semi-structured interviews.

**Results:**

Forty patients completed the study. All outcome criteria, quality of life (4.1±0.5 to 5.0±0.5; p<0.001), general symptoms (33.0±9.3 to 25.9±7.6; p<0.001) and the objective social situation (5.2±0.9 to 5.6±0.5; p=0.008) showed significant improvements. Patient reported largely positive experiences despite frequent technical problems with the online arrangements.

**Conclusions:**

The findings suggest that DIALOG+ is feasible and beneficial for patients with anxiety disorders in routine care and that a remote delivery is a feasible realistic option for administering it. Future research should assess implementation methods and effectiveness in larger controlled trials and identify the effective mechanisms in DIALOG+.

**Trial Registration:**

The trial was pre-registered (https://www.isrctn.com/ISRCTN38851969) on 16/12/2019.

## Background

Anxiety disorders are frequent, often recurrent or chronic and associated with high societal burden. Systematic reviews estimate the global point prevalence rate of anxiety disorders at 7.3% [1], and identify these disorders as the sixth leading cause of disability [2], in terms of years of life with disability. In order to reduce this burden worldwide, low-cost interventions are required that can be used in routine care and do not require setting up specialized services [3,4].

At the core of mental health care are routine meetings of patients and clinicians. Traditionally, such meetings are used to establish good relationships, assess problems, monitor symptoms and possibly discuss and arrange specific diagnostic or therapeutic interventions. In their communication clinicians may or may not use elements of psychological treatment approaches that had been developed and tested in different contexts, i.e., in specific psychological treatments and not in mainstream routine meetings.

DIALOG+ constitutes the first model that was specifically developed to make routine meetings therapeutically effective in themselves. Based on quality of life research, concepts of patient-centered communication and components of solution-focused therapy, it was developed in systematic research and shown to be effective in randomized controlled trials [5–7]. DIALOG+ is supported by an application. At the beginning, patients rating their satisfaction with eight life domains (mental health, physical health, job situation, accommodation, leisure activities, family relationships, friendships and personal safety), and three treatment aspects (medication, meetings with mental health professionals and practical help). The ratings range from 1 (‘totally dissatisfied’) to 7 (‘totally satisfied’), are displayed on the screen and can be compared with the ratings from any previous meeting. After a discussion of the overview, patients decide which life domain or treatment aspect they would like to discuss in the current meeting. Each area is then addressed in a four-step approach: 1) understanding (Why is the patient dissatisfied? What is nevertheless working well?); 2) looking forward (What is the best-case scenario? What is the smallest step forward?); 3) exploring options (What can the patient, the mental health professional and others do?); and finally, 4) agreeing on actions (What should be done between now and the next meeting?). The agreed actions are briefly documented and reviewed at the beginning of the next meeting.

The approach of DIALOG+ is rather generic and not diagnosis specific. Nevertheless, all the original research focused on patients with psychotic disorders [5,8] while later studies included also patients with mood disorders [6,9] and samples with severe mental illness and epilepsy [7]. However, there has been no research on using DIALOG+ with patients with anxiety disorders although anxiety disorders are a major challenge for mental health care world-wide.

Against this background, we aimed to conduct a non-controlled pilot trial of DIALOG+ with patients with anxiety disorders to assess experiences and outcomes of patients. After patient recruitment had already started, strict lockdowns because of the pandemic made in-person out-patient appointments impossible, and it was decided to provide DIALOG+ remotely.

## Materials and methods

### Study design

A pragmatic non-controlled pilot trial was conducted with patients with anxiety disorders using DIALOG+ over a six months period. The trial was conducted between May 2020 and March 2022. The research team trained mental health professionals in the use of DIALOG+, to be administered four times over a period of 6 months, as part of regular routine clinical meetings. The original plan to provide DIALOG+ every month over the six-month period was thought to be too intensive and therefore changed into four sessions with DIALOG+.

Data were collected at baseline and at the end of the six months intervention period.

### Participants and setting

Clinicians and patients were recruited from the CENECON – Center of Behavioral Neurology and Neuropsychiatry, of the School of Medicine of the University of Buenos Aires in Buenos Aires, Argentina. The Center receives most of the referrals from the Accident and Emergency Department and from other Departments of the Hospital of the School of Medicine. Normally, the majority of patients with mental disorders referred to that service suffer from anxiety disorders, and this group was regarded as a priority for testing new treatment options in the Center.

Clinicians were eligible if they had a qualification in any health care profession (e.g., psychiatrist, psychologist, nurse, occupational therapist, community mental health worker), more than one-month experience of working with patients with severe mental illnesses, and no plans to leave their post during the study period. Information was provided to potentially eligible professionals. The aim was to recruit at least five mental health professionals who met the inclusion criteria and would be assigned no more than 10 patients each.

The caseloads of recruited clinicians were screened to identify eligible patients. Patients had to meet the following inclusion criteria: aged 18 years or older; living in the community; a primary clinical diagnosis of anxiety disorders according to ICD-10 (F40-F48) [10]; and capacity to provide informed consent. Patients were excluded if they had learning disability or severe cognitive impairment, and if they had a baseline mean score of five or higher on the Manchester Short Assessment of Quality of Life (MANSA) [11]. This cut-off criterion on the MANSA was also used in other trials testing DIALOG+ [3,4,7], and is based on the assumption that, if the intervention aims to improve quality life, there is no point in including patients who already report a high level of quality of life. Given the exploratory nature of this pilot trial, the approach was pragmatic and avoided further restricting exclusion criteria. Participants were contacted by a researcher who provided information about the study. Patients who wanted to participate and provided written informed consent and completed the MANSA questionnaire.

### Procedure

Clinicians were trained in the use of DIALOG+ (Spanish version) by a member of the research team and received supervision sessions by senior clinicians on the study (NO, LB, FC), once every two months, after their DIALOG+ session. On 20^th^ March 2020, Argentina went into a lockdown due to the pandemic, making it impossible for out-patients to be seen at the hospital. At that time, patient recruitment to the study had already progressed, but DIALOG+ sessions had not yet started. In this situation, a decision was taken to continue with the study, but to deliver DIALOG+ remotely. The decision considered a) that DIALOG+ is supported by an App and that the displays used in the intervention can be shared on screen, so that the intervention lends itself to be delivered online; and b) that practically all patients have access to the internet through a computer or mobile.

All patient-clinician meetings at baseline and after 1, 2, 3 and 6 months were conducted remotely, using the Zoom platform with the screen sharing function, and a standardised approach for the remote delivery of DIALOG+ in the trial was developed and agreed on. The app, originally developed in English, was fully translated into Spanish to ensure cultural and linguistic appropriateness for the Argentine context. The Spanish version of the application was used throughout the study. Further details on the app and its features can be freely accessed on the internet [12,13].

After patients had signed the informed consent, and before starting the first DIALOG+ session, the clinician sent by e-mail a Zoom link to the patient and the connection was tested to make sure that it would work. The test was also used to explain to the patient how to use DIALOG+ through a shared screen.

During the sessions, patients and clinicians kept their cameras switched on, and the screen was shared from the tablet computer where the DIALOG+ application was installed. Patients rated their satisfaction aloud, and clinicians filled in the scores on the screens. For the rest of the session, the screen was shared if and when helpful. The recruitment period was from May 4^th^, 2020 to October 30^th^, 2021.

During the DIALOG+ study period, all patients continued to receive their usual treatment with the prescription of antidepressants and/or anxiolytics, if applicable.

### Measures

At baseline, patients and clinicians provided information on sociodemographic characteristics. For patients, the primary clinical diagnosis according to ICD-10 was obtained from the psychiatric medical records.

Three outcome criteria were assessed at baseline and at the end of the intervention period.

The primary outcome was subjective quality of life as assessed on the MANSA [11]. The MANSA includes 12 items on the patient’s satisfaction with life as a whole and 11 life domains, each of them rated between 1 (‘couldn’t be worse’) and 7 (‘couldn’t be better’). The mean score of those 12 items is taken as measure of quality of life.

Symptoms were observer-rated by two trained researchers on the 24-item version of the Brief Psychiatric Rating Scale (BPRS) [14]. Each symptom is assessed between 1 (‘not present’) and 7 (‘extremely severe’).

The objective social outcomes were assessed on the Social Outcomes Index (SIX) [15] which captures patient employment, accommodation and social contacts. SIX ranges from 0 (poorest social situation) to 6 (best social situation).

Untoward incidents were documented.

At the end of the study, a semi-structured interview was conducted with 15 patients to explore their experiences. The interview asked for positive and negative experiences, the perceived impact on the consultations, and barriers for using DIALOG+, and the sample included patients of both genders and all participating clinicians. Each interview was audio-recorded and transcribed verbatim.

### Sample size

Following the central limit theorem [16], we aimed to have a final sample for data analysis of 30 patients. Given the exploratory nature of this pilot trial, we allowed for a drop-out rate of 30% which required a baseline sample of 43 patients.

### Data analysis

For the statistical analysis, the SPSS program 27.0 was used. Changes of outcome criteria between baseline and end of intervention were analysed using paired t-tests.

An inductive content analysis was used to analyse patients’ statements in the interviews.

The study received ethics approval by the Alberto C. Taquini Institute for Research in Translational Medicine Biomedical Research Ethics Committee (IATIMET), School of Medicine, University of Buenos Aires, on 8^th^ February 2021.

## Results

**Fig 1:**
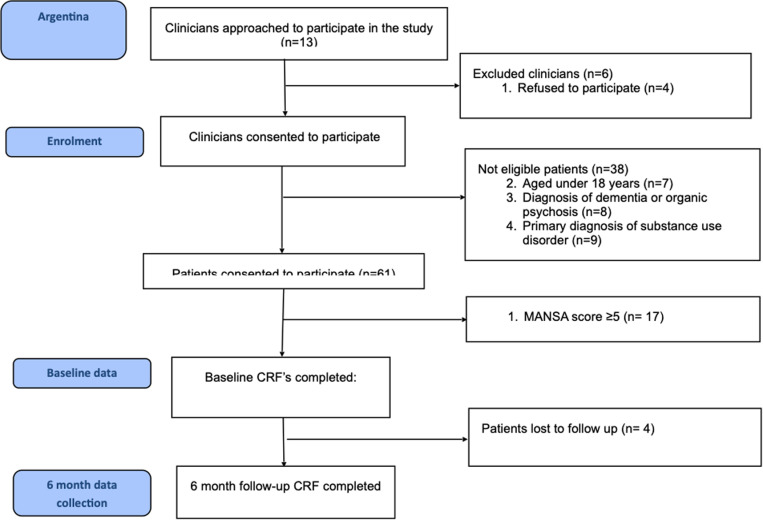
CONSORT participant flow diagram.

The recruitment and flow of clinicians and patients is shown in Fig 1.

Out of 13 clinicians invited to participate, seven consented. Five of them were psychiatry residents and two psychologists. They had a total caseload of 125 patients on the CENECON registry, and 61 met the inclusion criteria of age and clinical diagnosis and agreed to participate. After obtaining informed consent, 17 were excluded due to a MANSA score ≥ 5. Of the remaining 44 patients who started the study, three patients withdrew after one session from the study and one patient left the service and could not be contacted anymore. Thus, 40 patients completed the study. Each clinician had between 2 and 10 patients.

At the time of recruitment, 37 patients had been in treatment at the Center for between six and twelve months, two for two years and one for five years. Five patients did not receive any medication throughout the study period. At baseline, 32 patients were prescribed antidepressants and 26 tranquilizers. During the study period, doses were increased for four patients and decreased for four others, whilst the type of medication was modified for three patients.

All forty patients completed, as planned, four sessions of DIALOG+.

The characteristics of the patients are shown in Table 1.

**Table 1:** Baseline characteristics of the patients (n = 40).

	Total (n = 40)
Age: mean years (range)	38 (20–77)
Female (n, %)	27 (67.5%)
Primary psychiatric diagnosis	
F40.1 - Social anxiety disorder	1 (2.5%)
F41.0 - Panic disorder	9 (22.5%)
F41.1 - Generalized anxiety disorder	2 (5%)
F 41.1 - Anxiety disorder, unspecified	28 (70%)
Highest level of education	
Elementary	3 (7.5%)
Secondary	23 (57,5%)
Tertiary	3 (7.5%)
University/further education	11 (27.5%)
Employment status	
Employed full-time	15 (37.5%)
Employed part-time	12 (30%)
Unemployed	3 (7.5%)
Student	5 (12.5%)
Retired due to disability	1 (2.5%)
Retired on old age pension	2 (5%)
Supported by family pension	2 (5%)
Living situation	
Living alone	9 (22.5%)
Living with partner/family	31 (77.5%)
Marital status	
Single	17 (42.5%)
Married	6 (15%)
Co-habiting/civil partnership	11 (27.5%)
Separated	1 (2.5%)
Divorced	5 (12.5%)
Accommodation	
Independent accommodation	38 (95%)
With private support	1 (2,5%)
Homeless	1 (2,5%)

The mean age of the patients was 38 years (range 20–77). Most were women, and the most frequent primary diagnosis according to ICD-10 was anxiety disorder, unspecified (ICD-10: F 41.1).

### Outcomes

Scores of the three outcome measures at baseline and at six months are presented in Table 2.

**Table 2:** Outcomes at baseline and at the end of the six-month intervention period.

				Coefficient [95% CI]	p-value
		n	mean (SD)		
MANSA	Baseline	40	4.1 ± 0.5	…	…
	6 months	40	5.0 ± 0.4	-2.3 [-2.8 to -1.7]	<0.001
SIX	Baseline	40	5.2 ± 0.9	…	…
	6 months	40	5.6 ± 0.5	-0.4 [-0.7 to -0,07]	0.008
BPRS	Baseline	40	33.0 ± 9.3	…	…
	6 months	40	25.9 ± 7.6	0.9 [0.53–1.26]	<0.001

Outcomes on all three measures improved and all differences were statistically highly significant, with an effect size of 2.3 in the primary outcome of quality of life. This effect size reflects that the average score on the MANSA for the whole sample improved from 4.1 to 5.0. To achieve such a difference of 0.9 a patient must have improved, e.g., by one point of the seven-point scale in 10 out of the 12 included life domains or by five points in two life domains.

There was no untoward incident reported for any patient.

### Patient experiences

As planned, 15 interviews were conducted with patients after the six-month intervention.

Thirteen patients reported positive experiences of using DIALOG+ and felt that using it had a positive impact on their sessions with their clinicians. No patient reported negative effects. Two patients felt that using DIALOG+ had no real effect on the meetings with their clinicians without being explicitly critical.

“*I don’t think there was a change in my usual sessions after DIALOG, I think everything was fine.” (patient 2)*

The positive comments mentioned that DIALOG+ made patients think differently, that it had an empowering effect on decision making and that seeing progress over time was encouraging.

“*It was positive to see little progress from session to session. I wish I could continue using it.”(patient 8)*

“*The best thing that happened to me is that it helped me make decisions and make changes in my life that I couldn’t do because of the fears I had.” (patient 33)*

“*I felt comfortable using DIALOG. Having to think about how satisfied or dissatisfied I was with different aspects of my life and also having to rate them made me think in a very different way than I am used to. Especially thinking about my treatment or how my therapist can help me. I think it helped me a lot to understand what was happening to me and what actions to take.” (patient 18)*

“*With DIALOG I had to decide myself, be the one who said what had to be done and do it. Being able to see my progress after each session gave me more confidence in myself.” (patient 38)*

Patients in this study received DIALOG+ only as remote delivery and could not compare with an in-person provision. Only one patient commented on this and appreciated that the remote delivery made the sessions possible despite the pandemic.

“*I liked to participate. It was positive that it was remotely, because with the pandemic I couldn’t go to the hospital. It helped me to be able to think about the different aspects of my life and address each of them in an organized manner.” (patient 40)*

No patient mentioned the remote delivery as such as a potential barrier. However, eight of the 15 interviewed patients reported difficulties because of unreliable or failing internet connections.

“*What failed was the internet connectivity and the sessions were cut short.” (patient 38)*

## Discussion

### Main findings

The findings suggest that DIALOG+ as a relatively simple and low-cost intervention [5–9] can be used in routine meetings with patients with anxiety disorders, that it may be associated with relevant improvements, and that patient experiences are largely positive. Although not originally intended, the study also tested a remote delivery as there was no other option during the pandemic. Forty out of 44 patients completed the study, and all of them had the envisaged four meetings. These results also show that the remote use of DIALOG is feasible. Moreover, the identified pre-post changes suggest even this mode of delivery can have very positive outcomes, despite frequent problems with internet connections. The improvements were seen on different outcome criteria, i.e., on subjective quality of life as a self-rated measure, on observer-rated symptoms and on an objective index of the social situation. All of the improvements were large, statistically highly significant and clinically meaningful.

### Strengths and limitations

The study was conducted in routine patient-clinician meetings. It is the first study testing DIALOG+ with patients with a primary diagnosis of anxiety disorders, and also the first one in which DIALOG+ was exclusively delivered on-line. Although the research team had to develop the method of online delivery within a short period of time, the approach seems to have worked well, despite technical glitches. All eligible patients agreed to participate and about 90% of them completed the study, so that the sample was not selective and the study was pragmatic for the given context with wide inclusion criteria. The outcome measures reflected distinct criteria, and the effect sizes on all of them were clinically relevant.

However, the study also has limitations. Most importantly, the pilot trial was non-controlled. A randomized control group would have allowed to distinguish the improvements from potential spontaneous improvements during the study period. An active control – as used in some DIALOG+ trials [3] – could have gone even further and controlled also for the non-specific effects of attention and repeated ratings in the intervention. Secondly, there was no follow-up beyond the intervention period so that there is no information on the longer-term sustainability of the benefits. Thirdly, the study was conducted in only one service, and it remains unclear to what extent the results can be generalized to other services and other countries. Finally, the combination of two novelties, i.e., the first study of DIALOG+ with patients with anxiety disorders and the first study with consistent online use, makes it impossible to disentangle the effect of the two aspects, and one can only speculate as to whether an in-person delivery of DIALOG+ may have led to different results.

### Comparison with the wider literature

Our findings are consistent with the results of a randomized controlled trial of DIALOG+ in Bosnia and Herzegovina, where the sample was diagnostically mixed and included patients with depressive and anxiety disorders [4]. In that trial, DIALOG + led to improvements of quality of life and symptoms throughout a six-month intervention and after a further six-months follow-up period. The study had the same primary outcome of subjective quality of life with a medium effect size of 0.63. That effect size was substantially smaller than the one found in this non-controlled study but still statistically highly significant and clinically meaningful.

The large effect size in this study cannot directly be compared with effect sizes found in randomized controlled trials. Those trials compare changes in intervention and control groups and usually arrive at effect sizes that are substantially smaller [17]. In this study, the effect size considers pre-post changes in the intervention group without a control group. Systematic reviews and meta-analyses of such pre-post changes in psychological treatments of patients with anxiety disorders found average effect sizes of 1.22 [95%CI 1.14–1.30] [18] and 0.80 (95%CI 0.71–0.90) [19]. These effect sizes can be directly compared with the findings of our study. One can conclude that an effect size of more than 2 as achieved through DIALOG+ in this study can be considered as very positive when compared to the literature of related interventions. Moreover, when the potential benefits of DIALOG+ are compared against the effects of the psychological treatments included in those meta-analyses, one needs to consider that DIALOG+ was integrated in mainstream care and delivered as part of routine meetings. It does not require any referrals patients to specialized services and there is no need to have specifically qualified clinicians. DIALOG+ is low-cost and the findings of this study suggest that it can be used with a high proportion of patients in a given service. This may make a particularly attractive option for health care settings where both financial resources and qualified clinicians are scarce.

## Conclusions

The findings of this pilot trial are encouraging and suggest that routine meetings of clinicians with patients with anxiety disorders can be influenced and utilized so that they lead to relevant benefits. Further research should test both effectiveness and implementation in controlled trials, in larger samples and in a wider range of services [20]. Since DIALOG+ is an approach that can be flexibly applied in terms of frequency of use and duration of treatment, implementation should make use of this and go beyond a fixed schedule over a fixed period of time as applied in this study. Finally, a detailed research of treatment processes may reveal factors that can be improved in the delivery of DIALOG+ and potentially specified more for patients with anxiety disorders.

## Supporting information

S1 File Protocol. (PDF)

S2 File TREND Checklist. (PDF)

## Abbreviations

CI: Confidence Interval.
