# Supplementary material for: Outcomes and experiences of DIALOG+ provided remotely for patients with anxiety disorders—A non-controlled pilot trial
Source: PLoS One. 2025 May 19;20(5):e0321744. doi: 10.1371/journal.pone.0321744 (PMC12088059; doi:10.1371/journal.pone.0321744)
Supplement: S1 File — (PDF) [file pone.0321744.s001.pdf]

PROTOCOLO DE INVESTIGACIÓN

Estudio piloto de la intervención DIALOG+: Evaluando la factibilidad de implementar una intervención terapéutica para personas con problemas de salud mental en la Argentina

Investigadores Principales:

Stefan Priebe(Investigador Principal UK)

Luis Ignacio Brusco(Investigador Principal Argentina)

Versión:2.0

01 de febrero de 2021

INDICE

INDICE 2
1 INTRODUCCIÓN 3
2 OBJETIVOS DEL ESTUDIO 5
2.1 Objetivo Principal 5
2.2 Objetivos Secundarios 5
3 MEDICIÓN DE RESULTADOS Y OTRAS VARIABLES DEL ESTUDIO 6
3.1 Resultado Principal 6
3.2 Resultados Secundarios 6
4 DISEÑO DEL ESTUDIO 7
4.1 Diseño del Estudio 7
4.2 Ámbitos de Estudio 7
4.3 Participantes del Estudio 7
5 PARTICIPANTES 8
5.1 Descripción de los Establecimientos de Salud del Estudio 8
5.2 Criterios de Inclusión de los Participantes 8
5.3 Criterios de Exclusión de los Participantes 8
6 INTERVENCIÓN DEL ESTUDIO: DIALOG+ 10
7 PROCEDIMIENTOS Y EVALUACIONES DEL ESTUDIO 11
7.1 Reclutamiento 11
7.2 Entrenamiento y Supervisión de los Proveedores de Salud 11
7.3 Intervención 12
7.4 Seguimiento a los 6 meses 12
7.5 Entrevistas de salida 12
8 ANÁLISIS 13
9 ÉTICA Y PROTECCIÓN DE SUJETOS HUMANOS 14
9.1Aprobación Ética del Estudio 14
9.2 Aspectos Éticos y Regulatorios 14
9.3 Proceso de Consentimiento Informado 156
9.4 Confidencialidad de la Información 16
9.5 Riesgos Potenciales para los participantes 176
9.6 Beneficios Potenciales para los participantes 177
10 REFERENCIAS 188

1 INTRODUCCIÓN

En la Argentina, 1 de cada 3 personas presenta un problema de salud mental a partir de los 20 años[1]. En la Ciudad Autónoma de Buenos Aires (CABA), los trastornos neuropsiquiátricos son una importante causa de discapacidad,siendo la prevalencia por trastornos mentales, neurológicos y debidos al consumo de sustanciasdel 34% [2]. Pese a su alta prevalencia, existe una brecha de tratamiento entre 75% y 80%, siendo pocas las personas que reciben una atención en salud mental en el momento adecuado[3]. Estos trastornos suelen ser crónicos, requiriendo de intervenciones clínicas y comunitarias para mejorar la recuperación y calidad de vida del paciente.

Actualmente, la Argentina se encuentra en un proceso de reforma nacional a partir de la implementación de la Ley de Salud Mental N° 26.657, cuyo fundamento es fundamento es la perspectiva de Salud Mental Comunitaria e integración de los personas con padecimiento mental para la plena vigencia de sus derechos, conforme al principio de "no discriminación" [4]. A esta reforma se le suma la participación de las áreas programáticas con los Los centros de salud y acción comunitaria (CeSAC) de la CABA, las cuales son centros especializados en salud mental y que se encuentran articulados con los centro de atención primaria (CAP).Las áreas programáticas cuentan con equipos de profesionales de la salud, conformados por psiquiatra, psicólogo, médico de familia, enfermera, trabajador social, terapeuta ocupacional, terapeuta de lenguaje y técnicos de enfermería[5].

Existe evidencia de que una relación positiva entre proveedor-usuario es un factor predictor de mejoría a corto y largo plazo en pacientes que reciben atención psiquiátrica [6]. En este sentido, como parte del modelo de atención en salud mental comunitaria, se recomiendan las reuniones de rutina para evaluar los problemas de los usuarios, decidir sobre el tratamiento que seguirán y realizar un monitoreo de su progreso. Uno de los principales retos para la implementación de estas reuniones de rutina es el que sean terapéuticamente efectivasen sí mismas[7]. Por ello, un grupo de investigadores europeos ha desarrollado DIALOG+, la cual es una intervención que busca mejorar la comunicación proveedor-paciente y así los resultados del tratamiento de salud mental[8]. Esta intervención se basa en la investigación en calidad de vida, conceptos de comunicación centrada en el paciente, información tecnológica y componentes de la terapia centrada en soluciones, por medio de un aplicativo para Tablet o Smartphone.

DIALOG+ permite una valoración, planeamiento, intervención terapéutica y evaluación del paciente en un solo procedimiento, y requiere de un entrenamiento breve. Esta intervención ha sido testeada por medio de ensayos clínicos, mostrando resultados positivos en pacientes con psicosis, como mayores necesidades cubiertas, menos síntomas psicopatológicos y mejores resultados en su situación social objetiva[9, 10], además de presentar buenos resultados en los costos

asociados al tratamiento[8]. Es una intervención de bajo costo, que utiliza los recursos existentes de los servicios de salud y parece empoderar a los pacientes para que mejoren su condición de salud mental y situación social ya que centra en los recursos y potencialidades de sí mismos, sus familias y comunidad[10].

Se estima que DIALOG+ podría ser efectivo en países de bajos y medianos ingresos, donde los recursos suelen ser escasos para atender a las personas con trastornos mentales severos. Una buena estrategia es su implementación en los CeSAC, ya que aprovechará el trabajo que vienen realizando con este tipo de pacientes, y si mostrara buenos resultados, podría expandirse a nivel nacional.

2 OBJETIVOSDEL ESTUDIO

2.1 Objetivo Principal

Evaluar la implementación de la intervención DIALOG+para apoyar la atención en salud mental comunitaria e identificar mejoras en la calidad de vida de los usuarios a los 6 meses de haber iniciado la intervención.

2.2 Objetivos Secundarios

1) Analizar los cambios en los síntomas psiquiátricos de los usuarios participantes a los 6 meses de haber iniciado la intervención.
2) Analizar los cambios en la situación social objetiva de los usuarios participantes a los 6 meses de haber iniciado la intervención.
3) Explorar con los usuarios y proveedores de salud las barreras y facilitadores para la implementación de la intervención.
4) Recoger la experiencia de los usuarios en la intervención, explorando los beneficios para su vida diaria.
5) Recoger la experiencia de los proveedores en la intervención, explorando los beneficios para su atención a los usuarios con trastorno mental severo.

3 MEDICIÓNDE RESULTADOS Y OTRAS VARIABLES DEL ESTUDIO

Esta sección detalla los resultados principales y secundarios del estudio, las variables que serán recogidas a lo largo del mismo, los instrumentosque se usarán para medirlas, y el momento en que serán recolectadas.Además de las evaluaciones individuales a los participantes,a través de cuestionarios,para obtener los resultados principales y secundarios, también se recolectará información con los proveedores de salud y los participantespara evaluarla implementación de la intervención.

3.1 Resultado Principal

Mejora en la calidad de vida de los usuarios participantes, en base al puntaje de la escala Manchester Short Assessment of Quality of Life (MANSA), a los 6 meses de haber iniciado la intervención.

3.2 Resultados Secundarios

1) Mejora en los síntomas psiquiátricos de los usuarios participantes, en base al puntaje de la escala Brief Psychiatric Rating Scale (BPRS) a los 6 meses de haber iniciado la intervención.
2) Mejora en la situación social objetiva de los usuarios participantes, en base al puntaje de la escala Objective Social OutcomesIndex (SIX), a los 6 meses de haber iniciado la intervención.
3) Identificación de las barreras y facilitadores para la implementación de la intervención, por medio de entrevistas semi estructuradas a 10 usuarios y a todos los proveedores participantes, al finalizar el periodo de la intervención.
4) Experiencias positivas y negativas de los usuarios en la intervención y los beneficios para su vida diaria, por medio de entrevistas semi estructuradas a 10 usuarios participantes, al finalizar el periodo de la intervención.
5) Experiencias positivas y negativasde los proveedores en la intervención y los beneficios para su atención a los usuarios con trastorno mental severo, por medio de entrevistas semi estructuradas a 10 usuarios participantes, al finalizar el periodo de la intervención.

4 DISEÑO DEL ESTUDIO

4.1 Diseño del Estudio

Se realizará un estudio piloto de metodología mixta, en la cual se recogerán datos cuantitativos y cualitativos, de manera concurrente, esto es, en paralelo[11].

4.2 Ámbitos de Estudio

Las intervenciones se realizarán en forma remota, a través de una plataforma virtual (Zoom) debido a la situación de pandemia a raíz del virus SARS-CoV-2. Los proveedores de salud y usuarios serán reclutados a través del Centro de Neuropsiquiatría y Neurología Cognitiva (CENECON), de la Facultad de Medicina de la Universidad de Buenos Aires.

4.3 Participantes del Estudio

Usuarios de centros de salud mental comunitarios con trastorno de ansiedad y proveedores de salud de dichos establecimientos reclutados por el CENECON. En estos centros, los usuarios reciben atención especializada en salud mental con un modelo basado en la comunidad. Al ingresar al centro de salud mental comunitario son evaluados por un equipo interdisciplinario (psiquiatra, psicólogo y enfermera) quienes elaboran un Plan de Atención Individualizada junto con el usuario para determinar cómo será el tratamiento que recibirá. Este plan se va actualizando constantemente según los objetivos y necesidades de los usuarios.

Los proveedores de estos establecimientos han sido entrenados para brindar una atención basada en comunidad. Las actividades que realizan son tanto dentro como fuera del establecimiento de salud, incluyendo atención ambulatoria, talleres, visitas domiciliarias, entre otros. Asimismo, se organizan para trabajar de manera coordinada y así monitorear de manera conjunta a los usuarios, y articulan su trabajo con otros niveles de atención, dependiendo de las necesidades de sus usuarios (p. e. centros de salud del primer nivel de atención, hospitales).

La intervención se realizará con 40 usuarios de los centros de salud mental comunitarios y por lo menos 5 proveedores de salud, con no más de 10 usuarios cada uno.

5 PARTICIPANTES

5.1 Descripción de los Establecimientos de Salud del Estudio

El estudio se desarrollará en forma remota a través de la plataforma virtual Zoom.

5.2 Criterios de Inclusión de los Participantes

Todos los participantes del estudio deberán cumplir los siguientes criterios durante el proceso de reclutamiento:

Criterios de inclusión para los usuarios:

- Usuarios con diagnóstico primario de trastorno de ansiedad (CIE F40-F48),
- Tener 18 años o más
- Estar en capacidad de brindar consentimiento informado.
- Puntaje de 5 o menos en la escala Manchester Short Assessment of Quality of Life (MANSA).
- Recibir atención de alguno de los proveedores de salud participantesdel estudio.

Criterios de inclusión para los proveedores de salud:

- Profesional de salud que brinda atención clínica a los usuarios (ej. Psiquiatra, psicólogo, enfermera)
- Contar con un mes o más de experiencia trabajando con usuarios con trastornos de ansiedad .
- Trabajar actualmente en alguno de los centros de salud mental comunitarios participantes.
- No tener planes de dejar el centro de salud mental comunitario durante el periodo de implementación del estudio.

5.3 Criterios de Exclusión de los Participantes

Cualquier individuo que cumpla con los siguientes criterios durante el proceso de reclutamiento será excluido de participar en el estudio:

Criterios de exclusión para los usuarios:

- Diagnóstico de demencia o psicosis orgánica.
- Diagnóstico primario de trastorno por consumo de sustancias.
- Problemas severos de aprendizaje o discapacidad cognitiva severa.

Criterios de exclusión para los proveedores de salud:

- No tener contacto clínico con los usuarios.
- Tener contacto poco regular con los usuarios, por ejemplo, más de un mes entre cada contacto.
- Tener menos de un año de experiencia clinica

6 INTERVENCIÓN DEL ESTUDIO: DIALOG+

DIALOG+ es una intervención simple para evaluar la satisfacción del usuario con su vida y el tratamiento que vienen recibiendo, abordar las preocupaciones que tenga, y facilitar la comunicación entre el usuario y el proveedor de salud en la atención en salud mental. La intervención busca asegurar que la comunicación entre el usuario y el proveedor se centre en el usuario y que promueva un cambio positivo de manera efectiva.

La intervención se apoya en el uso de un aplicativo para tablets, en el cual se detallan todos los pasos a seguir en cada sesión con el usuario. En el aplicativo, el proveedor de salud puede agregar los usuarios participantes asignados, y crear nuevas sesiones por cada encuentro que tenga con ellos. En la primera sesión, el proveedor de salud compartirá a través de su pantalla el contenido de la tablet, explicando al usuario en qué consiste la intervención y dándole la oportunidad de familiarizarse con el procedimiento.

Cada sesión inicia con el usuario evaluando su satisfacción con ocho dominios de su vida (salud mental, salud física, situación laboral, vivienda, actividades recreativas/ocio, relación con la pareja y familia, amistades, seguridad personal) y con tres aspectos de su tratamiento (medicación, ayuda práctica, reuniones con los proveedores de salud). Cada dominio es puntuado en una escala que va del 1 (“totalmente insatisfecho”) al 7 (“totalmente satisfecho”). Dichas puntuaciones quedan registradas y pueden ser luego revisadas y comparadas con puntuaciones anteriores. Los proveedores de salud son instruidos de brindar retroalimentación positiva en caso de mejoras en la puntuación o de puntuaciones altas en los dominios.

Las puntuaciones son seguidas de una pregunta sobre si el usuario desea ayuda adicional con algún dominio. Con ayuda del proveedor de salud, el usuario elige algunos dominios para discutirlos con mayor detalle. Una vez seleccionados los dominios, se utiliza una metodología de 4 pasos centrado en la solución. Los cuatro pasos son: (1) comprender, consiste en conocer por qué el usuario se encuentra insatisfecho en ese dominio, y qué aspectos, a pesar de la insatisfacción, aún van bien en el dominio; (2) mirar hacia adelante, consiste en ayudar al usuario a identificar cuál es el escenario ideal y cuáles son los pasos más pequeños que puede tomar para llegar a dicho escenario); (3) considerar opciones, consiste en explorar e identificar qué puede hacer el usuario, el proveedor de salud y otras personas para lograr el cambio deseado; y (4) acordar tareas, consiste en llegar a un acuerdo sobre qué acción(es) debe(n) ser tomadas, y por quién. Luego de acordarse las acciones a seguir, éstas de registran en el aplicativo, y serán mostradas al inicio de la siguiente sesión para darles seguimiento.

7 PROCEDIMIENTOS Y EVALUACIONES DEL ESTUDIO

7.1 Reclutamiento

Los proveedores de salud serán reclutados en los centros de salud comunitarios donde laboran. A cada uno de ellos se les presentará el estudio y se explorará su interés en participar. Luego serán redireccionados al Centro de Neuropsiquiatría y Neurología Cognitiva (CENECON), de la Facultad de Medicina de la Universidad de Buenos Aires. De aceptar participar, se les pedirá firmar un consentimiento informado.

Los proveedores de salud, con ayuda del equipo de investigación, revisarán la carga de usuarios que atienden con el fin de identificar potenciales participantes que cumplan los criterios de inclusión. Se estima reclutar entre 5 a 10 usuarios por cada proveedor de salud. Los potenciales usuarios participantes serán contactados por el equipo de investigación para presentarles el estudio y explorar su interés en participar. A aquellos usuarios que acepten participar, se les pedirá firmar un consentimiento informado y completar el cuestionario MANSA para evaluar si son elegibles. Solo los usuarios con un puntaje igual o menor a 5 serán elegibles para continuar en el estudio.

A aquellos participantes que no son elegibles se les agradecerá por su tiempo y se les reembolsará sus gastos por refrigerio/conectividad .

Los participantes que sí son elegibles completarán el resto de la evaluación basal con el miembro del equipo de investigación. La evaluación basal consistirá, además del cuestionario MANSA, de 4 instrumentos más: un cuestionario sociodemográfico, un cuestionario sobre severidad de síntomas psiquiátricos (BPRS), un cuestionario sobre la situación social del usuario (SIX), y un cuestionario sobre su contacto con servicios de salud (CSRI). Una vez finalizado el cuestionario, se le agradecerá al usuario por su tiempo y se le reembolsará su gasto por refrigerio/conectividad .

7.2 Entrenamiento y Supervisión de los Proveedores de Salud

Los proveedores de salud que participen en el estudio recibirán una única sesión de entrenamiento (alrededor de 3 horas), brindada por el investigador principal de UK. En esta sesión se explicará el uso del aplicativo, y la metodología de la intervención.

Una vez iniciado el estudio, los proveedores participarán en una sesión de supervisión a las 4 semanas, en las que se absolverán dudas y reforzarán los contenidos brindados en el entrenamiento. Luego de ello, se realizarán sesiones de supervisión una vez cada dos meses, con sesiones adicionales a solicitud de los proveedores, de ser necesario.

Adicionalmente, durante las primeras semanas el equipo de investigación visitará a los proveedores para brindarles apoyo y absolver dudas, en caso sea necesario

7.3 Intervención

La intervención DIALOG+ será utilizada en las consultas entre el proveedor de salud y el usuario por un total de 6 meses, al inicio de manera mensual, y luego con menor frecuencia. En este sentido, la intervención será utilizada en la línea de base, al mes 1, 2, 3 y luego en el mes 6.

Cada proveedor tendrá asignado un número de participantes, y se les instruirá que solo apliquen la intervención DIALOG+ con ellos, esto con el fin de evitar que diferentes proveedores de salud apliquen la intervención con el mismo usuario participante, aumentando la frecuencia esperada de aplicación de la intervención.

7.4 Seguimiento a los 6 meses

Luego de 6 meses recibiendo la intervención DIALOG+, los usuarios participarán en una evaluación de seguimiento, donde un miembro del equipo de investigación les invitará a responder los mismos cuestionarios utilizados en la evaluación basal, a excepción del cuestionario sociodemográfico.

7.5 Entrevistas de salida

Adicionalmente al seguimiento de los usuarios, el equipo de investigación realizará entrevistas semiestructuradas con todos los proveedores de salud y una muestra de usuarios (25%, 10 en total). Estas entrevistas buscarán explorar los aspectos, tanto positivos como negativos, de la intervención, recoger las opiniones de los participantes y sus sugerencias para mejorar la intervención y su implementación. El audio de las entrevistas será grabado y transcrito literalmente.

8 ANÁLISIS

Se utilizarán estadísticos descriptivos para reportar la información sociodemográfica de los participantes. Para evaluar el impacto de la intervención, se calcularán y compararán las medias y las desviaciones estándar de las dos evaluaciones del estudio (evaluación basal y seguimiento). El resultado principal del estudio será la comparación de puntajes del cuestionario MANSA en el seguimiento de 6 meses, en comparación con la evaluación basal. Los datos del estudio serán analizados usando Stata para Windows (StataCorp, CollegeStation, TX).

Para analizar la información cualitativa obtenida en las entrevistas de salida, una persona externa al equipo de investigación se encargará de transcribir textualmente el audio de las entrevistas. Toda información que identifique al entrevistado será removida de la transcripción, incluyendo referencias a los usuarios o proveedores de salud.

El proceso de análisis iniciará con la creación de un libro de códigos basado en los temas principales identificados en las entrevistas. Todas las entrevistas serán analizadas utilizando el software Atlas.Ti (ATLAS.tiScientific Software DevelopmentGmbH, 2012).

9 ÉTICA Y PROTECCIÓN DE SUJETOS HUMANOS

9.1 Aprobación Ética del Estudio

El protocolo de investigación, los instrumentos y los consentimientos informados serán presentados para su revisión y aprobación por el Comité de Ética en Investigación Biomédica del Instituto Alberto C. Taquini de Investigaciones en Medicina Traslacional (IATIMET) de la Facultad de Medicina de la Universidad de Buenos Aires, y por el Comité de Ética de Queen Mary University of London antes de iniciar con el trabajo de campo.

9.2 Aspectos Éticos y Reulatorios

Este proyecto contempla la utilización de sujetos, por lo cual este ensayo debe realizarse deacuerdo con las Buenas Prácticas Clínicas (BPC). Antes de dar comienzo al proyecto el protocolo, el consentimiento informado y otros documentos delensayo deben contar con la aprobación de un Comité Independiente de Ética (CIE).Este comité debe formarse conforme a los requisitos reglamentarios pertinentes.Cuando corresponda, el CIE también deberá aprobar las enmiendas al protocolo antesde su ejecución en el centro, a menos que se justifique la ejecución anticipada paraeliminar un peligro inmediato. El CIE deberá emitir su aprobación por escrito y eldocumento deberá identificar de manera clara el ensayo, los documentos revisados(incluido el consentimiento informado) y la fecha de la revisión. Sólo se podrá poner enejecución el ensayo tal como está descripto en el protocolo (o la enmienda), firmar losconsentimientos informados y utilizar los documentos relativos al ensayo después deque se hayan obtenido todas las aprobaciones necesarias y es aceptable que elinvestigador comience con el ensayo.

Esta investigación se realizará bajo las normas regulatorias vigentes: Ley nacional25326 de Protección de Datos Personales de la República Argentina, Resolución1480/2011 del Ministerio de Salud de la República Argentina, Ley nacional 26529 deDerechos del Paciente en su Relación con los Profesionales e Instituciones de laSalud, y Ley 3301/09 de la Ciudad Autónoma de Buenos Aires de Protección deDerechos de Sujetos en Investigaciones en Salud. Los documentos y la normativainternacional a los que se ajusta esta investigación es la siguiente: el Informe Belmont,el código “Código” de Nuremberg, normas CIOMS/2012, declaración de Helsinsky.

El registro individual de los datos se hará en una base electrónica. El investigadordeberá proporcionar datos sobre los sujetos o los resultados de análisis obtenidossiguiendo las instrucciones acordes, conforme a las BPC.El investigador deberá llevar y conservar los registros y los datos durante el transcursodel ensayo conforme a todos los requisitos legales y reglamentarios pertinentes. Cadadato debe estar respaldado por un documento fuente que se encuentre en el centrodel

investigación. Todos los registros o documentos que se empleen como fuente deinformación (denominados “datos fuente del sujeto”) deben ser conservados para quepuedan examinarlos representantes autorizados del patrocinador o algún organismoregulador.

Es preciso completar un Registro de cada sujeto que otorgó su consentimientoinformado. No se deben recolectar datos personales como el nombre, las iniciales nininguna información personal de los sujetos que no sea necesaria para realizar elensayo. No está permitido identificar a los sujetos mediante el nombre ni las inicialesen el Registro ni en ningún otro documento del ensayo. La única información aceptableacerca de los sujetos que puede aparecer es el número único de identificación delsujeto. El investigador debe llevar una lista con información de contacto de cada sujetode manera que pueda comunicarse rápidamente con todos si fuera necesario.

.

9.3 Proceso de Consentimiento Informado

Antes de realizar cualquier procedimiento relacionado con el ensayo, es preciso enviar vía mail una descripción del protocolo en formato electrónico (pdf) a cada posible sujeto, explicarle detalladamente el protocolo y en qué consistirá su participación.

En el caso de los proveedores de salud, esto incluirá el entrenamiento, el uso de la intervención DIALOG+ durante las consultas con los usuarios y las entrevistas de salida. En el caso de los usuarios, esto incluirá la evaluación basal, la evaluación de seguimiento y la entrevista de salida.

Los miembros del equipo de investigación encargados del reclutamiento serán apropiadamente entrenados en cómo brindar la información necesaria a los potenciales participantes para obtener el consentimiento informado y cómo responder a sus preguntas.

Se asegurará a los participantes la naturaleza confidencial de toda la información provista, pero también se les informará que existen ciertos límites a la confidencialidad en situaciones en las que existe un riesgo serio para el participante o para otros. Bajo estas circunstancias, aun sin el consentimiento del participante, cierta información podría ser compartida con el centro de salud para garantizar su integridad o la de otras personas en riesgo.

También se les informará a todos los participantes que su participación es estrictamente voluntaria, y que la elección de no participar no tendrá ninguna consecuencia ni los afectará en ninguna forma. Los participantes del estudio no serán recompensados económicamente por su participación en la investigación, pero los costos de refrigerio/conectividad para las reuniones con el equipo de investigación serán reembolsados.

El participante podrá retirar su consentimiento en cualquier momento durante el estudio. Si esto ocurre, se consultará con el participante si la información recolectada hasta el momento del retiro de consentimiento puede ser utilizada o no. El equipo de investigación cumplirá con la decisión del participante al respecto.

El procedimiento de consentimiento informado se llevará a cabo en forma remota. . Las personas que cumplan los criterios de elegibilidad (usuarios y proveedores de salud) y estén interesadas en participar se les enviará el consentimiento informado vía e-mail, se coordinará una reunión con un miembro del equipo de investigación, y se programará una video-llamada entre los participantes y el investigador. En esta reunión, revisarán de manera conjuntan el documento de consentimiento informado y se responderá cualquier duda o consulta que surja. En el caso de personas que sean menores de 18 años, un padre o tutor legal puede estar presente en la reunión.

Si los participantes manifiestan querer ser parte del estudio, se les enviará un link para que accedan y puedan ver y revisar nuevamente en su pantalla el consentimiento informado. El consentimiento incluirá información clave: objetivos, procedimientos, registro y protección de datos, y riesgos y beneficios de participar. Luego se mostrarán tres preguntas adicionales al participante para verificar la comprensión de los puntos centrales del consentimiento. Finalmente, para consentir su participación deberá completar al final del documento sus datos (nombre, apellido, DNI) y marcar, virtualmente, un recuadro que certifique que han comprendido la finalidad y las condiciones del estudio y acepta voluntariamente participar. En el caso de los participantes menores de 18 años, este proceso será realizado en forma conjunta con su padre/madre o tutor legal. Ambos deberán contestar las preguntas y cargar los datos de filiación.

Estos consentimientos serán impresos por el equipo de investigación, se guardarán en la carpeta correspondiente y una vez que sea posible, se solicitará a cada participante que lo firme nuevamente con tinta.

9.4 Confidencialidad de la Información

Los participantes que acepten participar en el estudio recibirán un código de identificación que será utilizado a lo largo de su participación. Una lista de los códigos e información de identificación será guardada en una computadora protegida con contraseña, a la que solo tendrán acceso el equipo de investigación. De igual manera, durante las entrevistas los participantes tendrán la posibilidad de usar un seudónimo si así lo prefieren, con el fin de evitar mencionar sus nombres reales y queden registrados en las transcripciones.Por otro lado, las grabaciones de las entrevistas serán guardadas en una computadora protegida con contraseña que solo será accesible al equipo de investigación.

Los cuestionarios y consentimientos informados serán guardados, por separado, en gabinetes bajo llave en la oficina del equipo de investigación.

9.5 Riesgos Potenciales para los participantes

Los riesgos potenciales para los participantes de este estudio son mínimos, aunque se anticipan algunas circunstancias en que estos podrían manifestarse.

Durante la aplicación del cuestionario basal y de seguimiento, algunas preguntas pueden resultar incómodas para algunos participantes. Por ejemplo, para algunos, la naturaleza de las preguntas podríadespertar emociones como tristeza.El equipo de investigación encargado del reclutamiento y la aplicación de estos cuestionarios será entrenado para manejareste tipo de situaciones.

Asimismo, durante la aplicación de los cuestionarios, el equipo de investigación puede encontrarse con usuarios con riesgo suicida o que han sido víctimas de violencia. En estos casos, se buscará que el usuario reciba pronta atención por parte de los proveedores de salud disponibles en el centro de salud mental comunitario. Esto con el fin de garantizar la seguridad y el bienestar del usuario.

Algunos participantes podrían preocuparse por la confidencialidad de sus datos. Todos los participantes del estudio recibirán una explicación,incluida en el formato de consentimiento informado, sobre los procedimientos que se tomarán en cuenta para garantizar la confidencialidad de sus datos, entre los que se incluyen el uso de códigos en vez de nombres y el almacenamiento seguro de toda la información recolectada.

9.6 Beneficios Potenciales para los participantes

DIALOG+ es una intervención que complementa la atención que brindan los proveedores de salud, enfocando la atención brindada en las necesidades de los usuarios. En este sentido, se espera que la intervención tenga un impacto positivo en la comunicación usuario-proveedor.Asimismo, la intervención DIALOG+ ha probado ser efectiva en mejorar la calidad de vida y en reducir síntomas psiquiátricos en pacientes con psicosis, por lo que se espera que estos beneficios también se vean reflejados en los usuarios participantes del estudio.

Por último, la evidencia generada a través de esta investigación servirá como un primer insumo y evidencia para que a futurose pueda adaptar e implementar a mayor escala en más centros de salud mental comunitarios del Perú.

10 REFERENCIAS

1. Stagnaro JC1 et al. Twelve-month prevalence rates of mental disorders andservice use in the Argentinean Study of Mental Health Epidemiology. Soc Psychiatry Psychiatr Epidemiol. 2018 Feb;53(2):121-129. doi: 10.1007/s00127-017-1475-9.

2. Organización Mundial de la Salud. The Burden of Mental Disorders in the Region of the Americas, 2018 ISBN: 978-92-75-12028-6

3. Admisiones de salud mental en los hospitales del Gobierno de la Ciudad de Buenos Aires por sexo y grupo de edad según hospital. Ciudad de Buenos Aires. Años 2004/2015

4. Ley Nacional Nº 26.657 – Derecho a la Protección de la Salud Mental.

Decreto Reglamentario Nº 603/2013.

5. LEMUS, Jorge, y otros.- Las Comunas Y Su Impacto En La Regionalización Sanitaria De LaCiudad Autónoma De Buenos Aires.

6. McCabe R, Priebe S. The therapeutic relationship in the treatment of severe mental illness: a review of methods and findings. *Int J Soc Psychiatry* 2004;50(2):115-28.

7. Priebe S, McCabe R. The therapeutic relationship in psychiatric settings. *Acta Psychiatr Scand Suppl* 2006(429):69-72.

8. Priebe S, Golden E, Kingdon D, Omer S, Walsh S, Katevas K, et al. *Effective patient-clinician interaction to improve treatment outcomes for patients with psychosis: a mixed-methods design*. Southampton (UK); 2017.

9. Priebe S, Kelley L, Omer S, Golden E, Walsh S, Khanom H, et al. The Effectiveness of a Patient-Centred Assessment with a Solution-Focused Approach (DIALOG+) for Patients with Psychosis: A Pragmatic Cluster-Randomised Controlled Trial in Community Care. *Psychother Psychosom* 2015;84(5):304-13.

10. Omer S, Golden E, Priebe S. Exploring the Mechanisms of a Patient-Centred Assessment with a Solution Focused Approach (DIALOG+) in the Community Treatment of Patients with Psychosis: A Process Evaluation within a Cluster-Randomised Controlled Trial. *PLoS One* 2016;11(2):e0148415.

11. Creswell J, Piano Clark V. *Designing and Conducting Mixed Methods Research*. California: SAGE; 2007.

Translated from Spanish to English - www.onlinedoctranslator.com

RESEARCH PROTOCOL

Pilot study of the DIALOG+ intervention: Evaluating the feasibility of implementing a therapeutic intervention for people with mental health problems in Argentina

Principal researchers:

Stefan Priebe (Principal Investigator UK)

Luis Ignacio Brusco (Principal Researcher Argentina)

Version:2.0

February 1, 2021

INDEX

INDEX 2
1 INTRODUCTION 3
2 OBJECTIVES OF THE STUDY... 5
2.1 Main Objective... 5
2.2 Secondary Objectives... 5
3 MEASUREMENT OF RESULTS AND OTHER VARIABLES OF THE STUDY................. 6
3.1 Main Result... 6
3.2 Secondary Results... 6
4 STUDY DESIGN... 7
4.1 Study Design... 7
4.2 Areas of Study... 7
4.3 Study Participants 7
5 PARTICIPANTS... 8
5.1 Description of the Study Health Facilities 8
5.2 Participant Inclusion Criteria... 8
5.3 Participant Exclusion Criteria... 8
6 STUDY INTERVENTION: DIALOG+.. 10
7 STUDY PROCEDURES AND EVALUATIONS... 11
7.1 Recruitment... .... eleven
7.2 Training and Supervision of Health Providers.................................. 11
7.3 Intervention... 12
7.4 Follow-up after 6 months 12
7.5 Exit interviews 12
8 ANALYSIS 13
9 ETHICS AND PROTECTION OF HUMAN SUBJECTS... 14
9.1Ethical Approval of the Study 14
9.2 Ethical and Regulatory Aspects... 14
9.3 Informed Consent Process 156
9.4 Confidentiality of Information... 16
9.5 Potential Risks for Participants 176
9.6 Potential Benefits for Participants 177
10 REFERENCES... 188

1. INTRODUCTION

In Argentina, 1 in 3 people has a mental health problem from the age of 20[1]. In the Autonomous City of Buenos Aires (CABA), neuropsychiatric disorders are an important cause of disability, with the prevalence due to mental, neurological and substance use disorders being 34% [2]. Despite its high prevalence, there is a treatment gap between 75% and 80%, with few people receiving mental health care at the right time[3]. These disorders are usually chronic, requiring clinical and community interventions to improve the patient's recovery and quality of life.

Currently, Argentina is in a national reform process based on the implementation of Mental Health Law No. 26,657, whose foundation is the perspective of Community Mental Health and integration of people with mental illness for full validity. of their rights, in accordance with the principle of “non-discrimination” [4]. Added to this reform is the participation of the programmatic areas with theHealth and community action centers (CeSAC)of the CABA, which are centers specialized in mental health and that are articulated with the primary care centers (CAP). The programmatic areas have teams of health professionals, made up of a psychiatrist, psychologist, family doctor, nurse , social worker, occupational therapist, speech therapist and nursing technicians[5].

There is evidence that a positive provider-user relationship is a predictor of short- and long-term improvement in patients receiving psychiatric care [6]. In this sense, as part of the community mental health care model, routine meetings are recommended to evaluate users' problems, decide on the treatment they will follow, and monitor their progress. One of the main challenges for the implementation of these routine meetings is that they are therapeutically effective in themselves[7]. For this reason, a group of European researchers has developed DIALOG+, which is an intervention that seeks to improve provider-patient communication and thus the results of mental health treatment[8]. This intervention is based on research in quality of life, concepts of patient-centered communication, technological information and components of solution-focused therapy, through an application for Tablet or Smartphone.

DIALOG+ allows for patient assessment, planning, therapeutic intervention and evaluation in a single procedure, and requires brief training. This intervention has been tested through clinical trials, showing positive results in patients with psychosis, such as greater needs met, fewer psychopathological symptoms and better results in their objective social situation[9, 10], in addition to presenting good results in terms of costs.

associated with treatment[8]. It is a low-cost intervention that uses existing resources of health services and appears to empower patients to improve their mental health condition and social situation since it focuses on the resources and potential of themselves, their families and community. [10].

It is estimated that DIALOG+ could be effective in low- and middle-income countries, where resources are often scarce to care for people with severe mental disorders. A good strategy is its implementation in the CeSAC, since it will take advantage of the work they have been doing with this type of patients, and if it shows good results, it could expand nationally.

2 OBJECTIVES OF THE STUDY

2.1 Main goal

Evaluate the implementation of the DIALOG+ intervention to support community mental health care and identify improvements in the quality of life of users 6 months after starting the intervention.

2.2 Secondary Objectives

1) To analyze the changes in the psychiatric symptoms of the participating users 6 months after starting the intervention.
2) Analyze the changes in the objective social situation of the participating users 6 months after starting the intervention.
3) Explore with health users and providers the barriers and facilitators for the implementation of the intervention.
4) Collect the experience of users in the intervention, exploring the benefits for their daily lives.
5) Collect the experience of providers in the intervention, exploring the benefits for their care of users with severe mental disorders.

3 MEASUREMENT OF RESULTS AND OTHER STUDY VARIABLES

This section details the main and secondary results of the study, the variables that will be collected throughout it, the instruments that will be used to measure them, and the moment in which they will be collected. In addition to the individual evaluations of the participants, through questionnaires To obtain the main and secondary results, information will also be collected with health providers and participants to evaluate the implementation of the intervention.

3.1 Main Result

Improvement in the quality of life of the participating users, based on the Manchester Short Assessment of Quality of Life (MANSA) scale score, 6 months after starting the intervention.

3.2 Secondary Results

1) Improvement in the psychiatric symptoms of participating users, based on the Brief Psychiatric Rating Scale (BPRS) score 6 months after starting the intervention.
2) Improvement in the objective social situation of the participating users, based on the Objective Social OutcomesIndex (SIX) scale score, 6 months after starting the intervention.
3) Identification of barriers and facilitators for the implementation of the intervention, through semi-structured interviews with 10 users and all participating providers, at the end of the intervention period.
4) Positive and negative experiences of users in the intervention and the benefits for their daily lives, through semi-structured interviews with 10 participating users, at the end of the intervention period.
5) Positive and negative experiences of providers in the intervention and the benefits for their care of users with severe mental disorders, through semi-structured interviews with 10 participating users, at the end of the intervention period.

4 STUDY DESIGN

4.1 Study design

A mixed methodology pilot study will be carried out, in which quantitative and qualitative data will be collected, concurrently, that is, in parallel[11].

4.2 Study Areas

The interventions will be carried out remotely, through a virtual platform (Zoom) due to the pandemic situation resulting from the SARS-CoV-2 virus. Health providers and users will be recruited through the Center for Neuropsychiatry and Cognitive Neurology (CENECON), of the Faculty of Medicine of the University of Buenos Aires.

4.3 Study Participants

Users of community mental health centers with anxiety disorders and health providers from said establishments recruited by CENECON. In these centers, users receive specialized mental health care with a community-based model. Upon entering the community mental health center, they are evaluated by an interdisciplinary team (psychiatrist, psychologist and nurse) who develop an Individualized Care Plan together with the user to determine what the treatment they will receive will be. This plan is constantly updated according to the objectives and needs of users.

The providers at these facilities have been trained to provide community-based care. The activities they carry out are both inside and outside the health facility, including outpatient care, workshops, home visits, among others. Likewise, they are organized to work in a coordinated manner and thus jointly monitor users, and they articulate their work with other levels of care, depending on the needs of their users (eg first-level care health centers, hospitals).

The intervention will be carried out with 40 users of community mental health centers and at least 5 health providers, with no more than 10 users each.

5 PARTICIPANTS

5.1 Description of the Study Health Facilities

The study will be carried out remotely through the virtual platform Zoom.

5.2 Participant Inclusion Criteria

All study participants must meet the following criteria during the recruitment process:

Inclusion criteria for users:

- Users with a primary diagnosis of anxiety disorder (ICD F40-F48),
- to be 18 years old or more
- Be able to provide informed consent.
- Score of 5 or less on the Manchester Short Assessment of Quality of Life (MANSA) scale.
- Receive care from one of the health providers participating in the study.

Inclusion criteria for healthcare providers:

- Health professional who provides clinical care to users (e.g. psychiatrist, psychologist, nurse)
- Have a month or more of experience working with users with anxiety disorders.
- Currently working in one of the participating community mental health centers.
- Have no plans to leave the community mental health center during the study implementation period.

5.3 Participant Exclusion Criteria

Any individual who meets the following criteria during the recruitment process will be excluded from participating in the study:

Exclusion criteria for users:

- Diagnosis of dementia or organic psychosis.
- Primary diagnosis of substance use disorder.
- Severe learning problems or severe cognitive disability.

Exclusion criteria for healthcare providers:

- Do not have clinical contact with users.
- Have infrequent contact with users, for example, more than a month between each contact.
- Have less than one year of clinical experience

6 STUDY INTERVENTION: DIALOG+

DIALOG+ is a simple intervention to evaluate the user's satisfaction with their life and the treatment they have been receiving, address any concerns they have, and facilitate communication between the user and the health provider in mental health care. The intervention seeks to ensure that communication between user and provider is user-centered and effectively promotes positive change.

The intervention is supported by the use of an application for tablets, which details all the steps to follow in each session with the user. In the application, the health provider can add the assigned participating users, and create new sessions for each encounter they have with them. In the first session, the health provider will share the content of the tablet through their screen, explaining to the user what the intervention consists of and giving them the opportunity to become familiar with the procedure.

Each session begins with the user evaluating their satisfaction with eight domains of their life (mental health, physical health, work situation, housing, recreational/ leisure activities, relationship with their partner and family, friendships, personal safety) and with three aspects of their treatment (medication, practical help, meetings with health providers). Each domain is scored on a scale ranging from 1 (“completely dissatisfied”) to 7 (“completely satisfied”). These scores are recorded and can later be reviewed and compared with previous scores. Healthcare providers are instructed to provide positive feedback in case of score improvements or high domain scores.

The scores are followed by a question asking if the user would like additional help with any domain. With the help of the healthcare provider, the user chooses some domains to discuss in greater detail. Once the domains are selected, a 4-step solution-focused methodology is used. The four steps are: (1) understand, which consists of knowing why the user is dissatisfied in that domain, and what aspects, despite the dissatisfaction, are still going well in the domain; (2) looking forward, consists of helping the user identify what the ideal scenario is and what are the smallest steps they can take to get to that scenario); (3) considering options, consists of exploring and identifying what the user, the health provider and others can do to achieve the desired change; and (4) agreeing tasks, consists of reaching an agreement on what action(s) should be taken, and by whom. After agreeing on the actions to follow, these are recorded in the application, and will be shown at the beginning of the next session to track them.

7 STUDY PROCEDURES AND EVALUATIONS

7.1 Recruitment

Health providers will be recruited in the community health centers where they work. Each of them will be introduced to the study and their interest in participating will be explored. They will then be redirected to the Center for Neuropsychiatry and Cognitive Neurology (CENECON), of the Faculty of Medicine of the University of Buenos Aires. If you agree to participate, you will be asked to sign an informed consent.

Health providers, with the help of the research team, will review the load of users they serve in order to identify potential participants who meet the inclusion criteria. It is estimated to recruit between 5 to 10 users for each health provider. Potential participating users will be contacted by the research team to introduce them to the study and explore their interest in participating. Those users who agree to participate will be asked to sign an informed consent and complete the MANSA questionnaire to evaluate their eligibility. Only users with a score equal to or less than 5 will be eligible to continue in the study.

Those participants who are not eligible will be thanked for their time and will have their refreshment/connectivity expenses reimbursed.

Eligible participants will complete the remainder of the baseline assessment with the research team member. The baseline evaluation will consist, in addition to the MANSA questionnaire, of 4 more instruments: a sociodemographic questionnaire, a questionnaire on the severity of psychiatric symptoms (BPRS), a questionnaire on the user's social situation (SIX), and a questionnaire on their contact with services. of health (CSRI). Once the quiz is completed, the user will be thanked for their time and will be reimbursed for their snack/connectivity expense.

7.2 Training and Supervision of Health Providers

Healthcare providers participating in the study will receive a single training session (around 3 hours), provided by the UK principal investigator. In this session the use of the application and the methodology of the intervention will be explained.

Once the study has started, providers will participate in a supervision session after 4 weeks, in which doubts will be resolved and the content provided in the training will be reinforced. After that, supervision sessions will be held once every two months, with additional sessions at the request of providers, if necessary.

Additionally, during the first weeks the research team will visit the suppliers to provide support and answer questions, if necessary.

7.3 Intervention

The DIALOG+ intervention will be used in consultations between the health provider and the user for a total of 6 months, initially monthly, and then less frequently. In this sense, the intervention will be used at baseline, at month 1, 2, 3 and then at month 6.

Each provider will be assigned a number of participants, and they will be instructed to only apply the DIALOG+ intervention with them, this in order to prevent different health providers from applying the intervention with the same participating user, increasing the expected frequency of application of the intervention. intervention.

7.4 Follow-up after 6 months

After 6 months receiving the DIALOG+ intervention, users will participate in a follow-up evaluation, where a member of the research team will invite them to answer the same questionnaires used in the baseline evaluation, with the exception of the sociodemographic questionnaire.

7.5 Exit interviews

In addition to monitoring users, the research team will conduct semi-structured interviews with all health providers and a sample of users (25%, 10 in total). These interviews will seek to explore the aspects, both positive and negative, of the intervention, collect the opinions of the participants and their suggestions to improve the intervention and its implementation. The audio of the interviews will be recorded and transcribed verbatim.

8 ANALYSIS

Descriptive statistics will be used to report the sociodemographic information of the participants. To evaluate the impact of the intervention, the means and standard deviations of the two study evaluations (baseline evaluation and follow-up) will be calculated and compared. The main outcome of the study will be the comparison of MANSA questionnaire scores at the 6-month follow-up, compared to the baseline evaluation. Study data will be analyzed using Stata for Windows (StataCorp, CollegeStation, TX).

To analyze the qualitative information obtained in the exit interviews, a person external to the research team will be in charge of transcribing the audio of the interviews verbatim. All information that identifies the interviewee will be removed from the transcript, including references to health users or providers.

The analysis process will begin with the creation of a codebook based on the main themes identified in the interviews. All interviews will be analyzed using the Atlas.Ti software (ATLAS.tiScientific Software DevelopmentGmbH, 2012).

9 ETHICS AND PROTECTION OF HUMAN SUBJECTS

9.1 Ethical Approval of the Study

The research protocol, instruments and informed consents will be presented for review and approval by the Biomedical Research Ethics Committee of the Alberto C. Taquini Institute for Research in Translational Medicine (IATIMET) of the Faculty of Medicine of the University of Buenos Aires. Aires, and by the Ethics Committee of Queen Mary University of London before beginning field work.

9.2 Ethical and Regulatory Aspects

This project contemplates the use of subjects, which is why this trial must be carried out in accordance with Good Clinical Practices (GCP). The protocol, informed consent and other trial documents must be approved by an Independent Ethics Committee (IEC) before the project begins. This committee must be formed in accordance with relevant regulatory requirements. Where applicable, the IEC must also approve amendments to the protocol before execution at the center, unless early execution is justified to eliminate an immediate danger. ICN must issue its approval in writing and the document must clearly identify the trial, the documents reviewed (including informed consent), and the date of the review. The trial as described in the protocol (or amendment), signed informed consents, and use of documents relating to the trial may only be carried out after all necessary approvals have been obtained and it is acceptable for the investigator to begin the trial.

This research will be carried out under current regulatory standards: National Law 25326 on the Protection of Personal Data of the Argentine Republic, Resolution 1480/2011 of the Ministry of Health of the Argentine Republic, National Law 26529 on Patient Rights in their Relationship with Health Professionals and Institutions , and Law 3301/09 of the Autonomous City of Buenos Aires on the Protection of Rights of Subjects in Health Research. The documents and international regulations to which this research conforms are the following: the Belmont Report, the Nuremberg “Code”, CIOMS/2012 standards, Helsinki declaration.

The individual registration of data will be done in an electronic database. The investigator must provide data on subjects or test results obtained by following appropriate instructions, in accordance with GCP. The investigator must maintain and maintain records and data during the course of the trial in accordance with all relevant legal and regulatory requirements. Each data must be supported by a source document that is at the center of the

investigation. All records or documents used as sources of information (referred to as "subject source data") must be retained for review by authorized representatives of the sponsor or a regulatory body.

A Registry must be completed for each subject who gave informed consent. Personal data such as name, initials or any personal information of the subjects that is not necessary to carry out the trial should not be collected. Identification of subjects by name or initials is not permitted in the Registry or in any other trial document. The only acceptable information about subjects that can appear is the subject's unique identification number. The researcher should keep a list of contact information for each subject so that he can quickly contact everyone if necessary.

.

9.3 Informed Consent Process

Before carrying out any procedure related to the trial, it is necessary to send a description of the protocol in electronic format (pdf) via email to each potential subject, explaining the protocol in detail and what their participation will consist of.

For healthcare providers, this will include training, use of the DIALOG+ intervention during client consultations, and exit interviews. For clients, this will include the baseline assessment, follow-up assessment, and exit interview.

Recruiting research team members will be appropriately trained in how to provide the information necessary to potential participants to obtain informed consent and how to respond to their questions.

Participants will be assured of the confidential nature of all information provided, but will also be informed that there are certain limits to confidentiality in situations where there is a serious risk to the participant or others. Under these circumstances, even without the participant's consent, certain information could be shared with the health center to guarantee its integrity or that of other people at risk.

All participants will also be informed that their participation is strictly voluntary, and that choosing not to participate will have no consequences or affect them in any way. Study participants will not be financially compensated for their participation in the research, but refreshment/connectivity costs for meetings with the research team will be reimbursed.

The participant may withdraw his or her consent at any time during the study. If this occurs, the participant will be consulted whether the information collected up to the moment of withdrawal of consent can be used or not. The research team will comply with the participant's decision in this regard.

The informed consent procedure will be carried out remotely. . People who meet the eligibility criteria (users and health providers) and are interested in participating will be sent informed consent via e-mail, a meeting will be coordinated with a member of the research team, and a video call will be scheduled. between the participants and the researcher. At this meeting, they will jointly review the informed consent document and any questions or queries that may arise will be answered. For individuals who are under 18 years of age, a parent or legal guardian may be present at the meeting.

If the participants state that they want to be part of the study, they will be sent a link so that they can access it and be able to see and review the informed consent again on their screen. The consent will include key information: objectives, procedures, registration and data protection, and risks and benefits of participating. Three additional questions will then be shown to the participant to check understanding of the core points of consent. Finally, to consent to your participation, you must complete your information at the end of the document (name, surname, ID) and virtually mark a box certifying that you have understood the purpose and conditions of the study and voluntarily agree to participate. In the case of participants under 18 years of age, this process will be carried out jointly with their father/mother or legal guardian. Both must answer the questions and upload the affiliation data.

These consents will be printed by the research team, kept in the appropriate folder, and once possible, each participant will be asked to sign it again in ink.

9.4 Confidentiality of information

Participants who agree to participate in the study will receive an identification code that will be used throughout their participation. A list of the codes and identifying information will be saved on a password-protected computer, to which only the research team will have access. Likewise, during the interviews, participants will have the possibility of using a pseudonym if they prefer, in order to avoid mentioning their real names and they will be recorded in the transcripts. On the other hand, the recordings of the interviews will be saved in a password protected computer that will only be accessible to the research team.

The questionnaires and informed consents will be kept, separately, in locked cabinets in the research team's office.

9.5 Potential Risks for Participants

The potential risks for the participants in this study are minimal, although some circumstances in which these could manifest are anticipated.

During the administration of the baseline and follow-up questionnaire, some questions may be uncomfortable for some participants. For example, for some, the nature of the questions could arouse emotions such as sadness. The research team in charge of recruiting and administering these questionnaires will be trained to handle these types of situations.

Likewise, during the application of the questionnaires, the research team may encounter users at risk of suicide or who have been victims of violence. In these cases, the user will be sought to receive prompt attention from the health providers available at the community mental health center. This in order to guarantee the safety and well-being of the user.

Some participants may be concerned about the confidentiality of their data. All study participants will receive an explanation, included in the informed consent form, of the procedures that will be taken to ensure the confidentiality of their data, including the use of codes instead of names and secure storage. of all the information collected.

9.6 Potential Benefits for Participants

DIALOG+ is an intervention that complements the care provided by health providers, focusing the care provided on the needs of users. In this sense, it is expected that the intervention will have a positive impact on user-provider communication. Likewise, the DIALOG+ intervention has proven to be effective in improving quality of life and reducing psychiatric symptoms in patients with psychosis, so it is expected that these benefits are also reflected in the users participating in the study.

Finally, the evidence generated through this research will serve as a first input and evidence so that in the future it can be adapted and implemented on a larger scale in more community mental health centers in Peru.

10 REFERENCES

1. Stagnaro JC1 et al. Twelve-month prevalence rates of mental disorders and service use in the Argentinean Study of Mental Health Epidemiology. Soc Psychiatry Psychiatr Epidemiol. 2018 Feb;53(2):121-129. doi: 10.1007/s00127-017-1475-9.

2. World Health Organization. The Burden of Mental Disorders in the Region of the Americas, 2018 ISBN: 978-92-75-12028-6

3. Mental health admissions in the hospitals of the Government of the City of Buenos Aires by sex and age group according to hospital. Buenos aires city. Years 2004/2015

4. National Law No. 26,657 – Right to Protection of Mental Health.

Regulatory Decree No. 603/2013.

5. LEMUS, Jorge, and others.- The Communes and Their Impact on the Health Regionalization of the Autonomous City of Buenos Aires.

6. McCabe R, Priebe S. The therapeutic relationship in the treatment of severe mental illness: a review of methods and findings.*Int J Soc Psychiatry* 2004;50(2):115-28.

7. Priebe S, McCabe R. The therapeutic relationship in psychiatric settings.*Acta Psychiatr Scand Suppl*2006(429):69-72.

8. Priebe S, Golden E, Kingdon D, Omer S, Walsh S, Katevas K, et al.*Effective patient-clinician interaction to improve treatment outcomes for patients with psychosis: a mixed-methods design*. Southampton (UK); 2017.

9. Priebe S, Kelley L, Omer S, Golden E, Walsh S, Khanom H, et al. The Effectiveness of a Patient-Centred Assessment with a Solution-Focused Approach (DIALOG+) for Patients with Psychosis: A Pragmatic Cluster-Randomised Controlled Trial in Community Care.*Psychother Psychosom*2015;84(5):304-13.

10. Omer S, Golden E, Priebe S. Exploring the Mechanisms of a Patient-Centred Assessment with a Solution Focused Approach (DIALOG+) in the Community Treatment of Patients with Psychosis: A Process Evaluation within a Cluster-Randomised Controlled Trial.*PLoS One*2016;11(2):e0148415.

11. Creswell J, Piano Clark V.*Designing and Conducting Mixed Methods Research*. California: SAGE; 2007.
